# Commentary: Cognitive, Emotional, and Psychosocial Functioning of Girls Treated with Pharmacological Puberty Blockage for Idiopathic Central Precocious Puberty

**DOI:** 10.3389/fpsyg.2017.00044

**Published:** 2017-01-23

**Authors:** Peter Hayes

**Affiliations:** Faculty of Education and Society, University of SunderlandSunderland, UK

**Keywords:** GnRHa, precocious puberty, early puberty, IQ, cognitive performance

Gonadotropin releasing hormone agonists (GnRHas) have been found to impair memory in adults, so the study by Wojniusz et al. (2016) on the possible cognitive effects of these drugs on children treated for idiopathic central precocious puberty (CPP) represents an important contribution to research in this area. Recent findings that GnRHas increase depression symptoms (Macoveanu et al., 2016) and slow reaction time (Stenbæk et al., 2016) in healthy women, and reduce long-term spatial memory in sheep (Hough et al., 2017) underline the importance of the research that Wojniusz et al. (2016) have undertaken. However, their reassuring statement in the abstract that girls undergoing GnRHa treatment for CPP and controls “showed very similar scores with regard to cognitive performance” and their conclusion that “GnRHa treated girls do not differ in their cognitive functioning … from the same age peers” (Wojniusz et al., 2016) may be overly optimistic. These statements minimize the fairly substantial difference found in IQ scores and may also overemphasize its lack of statistical significance, as given the small number of participants in the study statistical significance has a high threshold. The statements should be qualified to indicate that the research has, in fact, reinforced concerns over the impact of GnRHas on cognitive performance in children.

Girls treated for CPP with triptorelin acetate were tested with the short form Wechsler Intelligence Scale for Children III. It was found that the girls had a mean IQ of 94, as against a mean IQ of 102 for the matched control group (Wojniusz et al., 2016). These IQ estimations are presented as standardized IQ scores, which places a girl scoring 102 at the 55th percentile, and a girl scoring of 94 at the 34th percentile. It is questionable whether scores that indicate a percentile gap of this size can be described as “very similar.” The 8 point gap is not statistically significant (*p* = 0.09) but, as the authors point out, this may be a function of the small number of participants (15 treated girls, 15 controls).

The authors contend that despite the small number of participants the results can—probably—be relied on to indicate that if GnRHas *do* cause a decline in IQ, this decline will be under 1 standard deviation (*SD*), which “represents a boundary of what is a clinically interesting difference” (Wojniusz et al., 2016). The contention that a decline only becomes clinically interesting if it is of at least 1 standard deviation is unconvincing. Any findings which indicate that GnRHas cause a decline, even a modest decline, in IQ are likely to be of considerable interest to patients and their parents. It is a factor that they may well want to consider in deciding whether or not to take the drug. They may, for example, wish to consider the possible effect of GnRHas on a child's school and exam performance. In this respect it can be noted that 2 of the treated girls had been held back a year at school. Given their advanced physical maturity, children with precocious puberty may find it particularly uncomfortable to be put in a class where they are a year older than their class mates. If GnRHa treatment does cause a reduction in IQ, this may contribute to the decision to place a child in a lower age year group. Certainly, treatment that has a deleterious effect on IQ will do nothing to help children who are academically behind to catch up.

The question of whether a drop in IQ of around 8 points has clinical significance must also be considered in the context of the uncertain benefits of GnRHa treatment for CPP. The ability of GnRHas to increase final height has not been confirmed by randomized controlled trials (Bouvattier et al., 1999; Cassio et al., 1999). Where girls with CPP experience psychosocial difficulties, providing support rather than drugs may be the most appropriate response (Hayes, 2016).

The findings of Wojniusz et al. (2016) can be compared with those of a 2001 study in which 25 children treated for early puberty with triptorelin acetate were tested with the short form Wechsler Intelligence Scale for Children (Mul et al., 2001). In this longitudinal study, children took the IQ test before treatment and again after 2 years of treatment. It was found that their IQ dropped 7 points from 100 to 93. With 25 treated participants, this 7 point drop was significant (*p* = 0.002). In both studies the difference in the performance element of the test was greater than in the verbal element. The similarities between the findings of these two studies strengthens their reliability and increases the possibility that GnRHa treatment may have an adverse impact on cognitive functioning in children. This makes it yet more important for further research to be carried out into the effects GnRHas may have on cognitive performance in children.

## Author contributions

The author has read and analyzed the published data on which the manuscript is based, written the manuscript and reviewed the manuscript.

### Conflict of interest statement

The author declares that the research was conducted in the absence of any commercial or financial relationships that could be construed as a potential conflict of interest.
